# Handcuffed for 15 min: public perceptions of restraint and seclusion in schools: an experimental study of race and disability

**DOI:** 10.3389/fpsyg.2025.1646644

**Published:** 2026-01-23

**Authors:** Da'Shay Templeton, Ruslan Korchagin

**Affiliations:** California Lutheran University, Thousand Oaks, CA, United States

**Keywords:** seclusion and restraint of minors, online survey experiment, DisCrit theory, QuantCrit, racism, abelism, school discipline, disability

## Abstract

**Introduction:**

This study examines how members of the U.S. public evaluate the use of restraint and seclusion in schools when the student’s disability and racial identities vary. Restraint and seclusion are legally designated as emergency safety interventions; yet, they are disproportionately used on disabled students, particularly those who are also racially marginalized. Drawing on Disability Critical Race Theory (DisCrit), this study frames these practices as situated at the intersection of racism and ableism.

**Methods:**

Methodologically, the study adopts a QuantCrit approach through a randomized online survey experiment with six experimental conditions that varied the students’ race (White, Black, or American Indian) and disability status (disabled or non-disabled). Participants rated school personnel performance, whether the student deserved punishment, whether the punishment fit the behavior, and whether the incident was prejudiced.

**Results:**

Results indicate that disability status, rather than race, significantly shaped participants’ evaluations. Across scenarios, non-disabled students were more likely to be viewed as deserving of punishment and as having received punishment that fit the behavior. Participants also showed slightly higher perceptions of prejudice when the student was Black than when the student was White.

**Discussion:**

These findings suggest a need to further examine how disability is interpreted in public judgments of school discipline and to pursue policy reforms that reduce reliance on restraint and seclusion.

## Introduction

In 2014, an 8-year-old boy with post-traumatic stress disorder and attention deficit hyperactivity disorder—who was later identified as a student of color—was handcuffed above the elbows for striking at school personnel at Latonia Elementary School in Kentucky (Mizner, 2018). On 11 November, he exhibited violent behavior against school personnel who physically restrained him to keep him from kicking them. After the incident, he was suspended for 1 day. On November 13th, when he returned to school, he exhibited similar behavior, but this time, the principal contacted a sworn law enforcement officer. The high-profile case captured national attention and concern when a video of the boy screaming and crying went viral (ACLU, 2024).

The sworn law enforcement officer (SLEO) who handcuffed the boy for 15 min was charged with unreasonable seizure and excessive force (S.R., et al. v. Kenton County Sheriff’s Office, 2017). Sadly, this incident of restraint and seclusion is far from unique. This case received widespread attention because video documentation made the incident publicly visible. In fact, in 2013–14, the Civil Rights Data Collection found that “more than 100,000 students were placed in seclusion or involuntary confinement or were physically restrained at school to immobilize them or reduce their ability to move freely—including almost 69,000 students with disabilities” (CRDC, 2014, p. 5). School personnel underreport instances of restraint and seclusion, so these estimates could be greater (Knackstedt, 2017). There is limited transparency regarding the use of restraint and seclusion in schools, a concerning issue given that many disabled students may be unable to report such experiences due to communication barriers (Butler, 2019). Restraint and seclusion are legally designated as emergency safety interventions intended for use only when there is an imminent risk of serious physical harm. However, research and federal monitoring systems show that these practices are sometimes used in situations that do not meet this legal threshold, and their use can function effectively as a disciplinary response. This study brings incidents of restraint and seclusion to the general U.S. public through an online experiment that collects their opinions on these harsh practices. This experimental research provides insight into public perceptions that may inform ongoing policy discussions regarding restraint, seclusion, and the role of school-based law enforcement.

The role of SLEOs and school resource officers (SROs) becomes increasingly problematic because of a general lack of regulation related to SLEOs and SROs, as well as seclusion and restraint (Butler, 2019; Ryan et al., 2018). Yet, 24% of elementary schools have SLEOs and SROs (CRDC, 2014)—with that number continually increasing (Haag, 2021). Restraint and seclusion are nationwide practices; yet, “there is no federal law comprehensively regulating the use of restraint and seclusion in schools” (Butler, 2019, p. x). State legislation governing restraint and seclusion varies widely in definitions, training requirements, reporting procedures, and permitted practices, resulting in uneven protections for students across the country (Kern et al., 2024; Graves, 2023). Though the literature on school discipline is vast, academic scholarship rarely covers restraint and seclusion, let alone captures public opinions of them. This article argues that under the regime of White supremacy, harsh school discipline practices negatively impact the academic and life outcomes of disabled students, especially along racial lines.

Using a QuantCrit approach, the study explores how the U.S. public justifies seclusion and restraint against disabled Black boys and disabled American Indian boys. QuantCrit is a quantitative methodological approach underpinned by critical theories such as DisCrit. QuantCrit research is critical because prior research shows that restraint and seclusion are used disproportionately on disabled students, Black students, and American Indian students (Butler, 2019), which necessitates an investigation of the intersection of ableism and racism.

The public influences educational policy in America. For example, following public outcry after mass shootings, educational policy reforms resulted in increased police presence in schools and zero-tolerance policies and, to a lesser extent, anti-bullying policies, emergency management planning, peer mediation, and school climate policies (Curran et al., 2020; Muschert and Peguero, 2010). As such, the study examines how the public rates the general performance of teachers, principals, and SLEOs who restrain, seclude, and suspend students, given the public’s tremendous influence on public policy (Burstein, 2003). And yet, their opinions on restraint and seclusion have not been explored in research. This study advances our understanding of how disability and race intersect in public attitudes toward restraint and seclusion, drawing on DisCrit to frame how social categories shape perceptions of discipline in schools.

## Literature review

### Race, disability, and the use of restraint and seclusion in schools

Ableism and racism are systems of oppression with various categories that are socially constructed and operate under a regime of white supremacy (Bell, 1980; Smith, 2004). In terms of racial categories, the study uses the term Black to capture the experience of the Black diaspora in American schools. Likewise, American Indian captures the experiences of Indigenous populations into America. In terms of disability categories, the study divides non-disabled and disabled populations in two groups for the sake of the experimental design. However, the study recognizes that race and disability are fluid and non-binary. For the purposes of this study, disability is conceptualized through a social model in which exclusionary structures and institutional arrangements create disabling conditions, rather than locating disability within individual bodies. In this article, the study uses identity-first language (e.g., “disabled student”) rather than person-first language (e.g., “student with a disability”). Identity-first language aligns with disability justice movements that view disability as a valued identity and sociopolitical category rather than a deficit to be minimized (Annamma et al., 2013). While person-first language is more commonly used in K–12 education settings, identity-first language reflects how many disabled scholars, activists, and communities describe themselves and aligns with a DisCrit framing that acknowledges disability as constructed through social, historical, and institutional forces rather than as an inherent individual impairment. These constructs have material consequences in schools, particularly in discipline practices such as restraint and seclusion.

Since the 18^th^ century, psychiatric and hospital staff have used physical restraints to subdue patients. Since the 1950s, school personnel have done the same (Ryan and Peterson, 2004). According to the CRDC (2019), “physical restraint is a personal restriction that immobilizes or reduces the ability of a student to move his or her torso, arms, legs, or head freely. . . .Seclusion is the involuntary confinement of a student alone in a room or area from which the student is physically prevented from leaving” (p. 4). Although mechanical restraints have been documented (Butler, 2019), the most commonly used restraint procedures in U.S. schools are physical, manual restraints that involve staff immobilizing a student’s movement. Increasingly, restraint and seclusion have made the education policy agenda at the state and federal levels. For example, the Hartford Courant investigated and documented hundreds of deaths of students—most of whom were disabled—due to restraint and seclusion more than 20 years ago (Butler, 2019). Then, in 2009, Congress began focusing on the restraint and seclusion of children with disabilities. Since then, federal legislation—meant to provide greater safeguards for restraint and seclusion—has been introduced at each session of the U.S. Congress. Yet these and other state legislations have not progressed.

Restraint and seclusion practices disproportionately impact vulnerable populations, primarily disabled students of color (Butler, 2019; CRDC, 2019). Research conclusively shows that discrimination, coupled with broadly discretionary zero-tolerance discipline policies, is violating the civil rights of disabled students of color. Beyond civil rights violations, restraint and seclusion lead to trauma, injury, and even death (Kutz, 2009; NDRN, 2012). Moreover, many agencies—medical, psychiatric, law enforcement, etc.—that use physical restraint are trained to do so because of strict guidelines, including accreditation requirements from multiple governing agencies like the Joint Commission on Accreditation of Healthcare Organizations, the American Academy of Pediatrics, the National Association of Psychiatric Treatment Centers for Children, and others (Ryan and Peterson, 2004). In contrast, schools lack these requirements and oversight, which leads to more instances of injury, trauma, and death (Ryan and Peterson, 2004). Moreover, there are multiple inconsistencies in implementation at state and local levels (NDRN, 2012), including the purview of SLEOs and SROs (Ryan et al., 2018). Protections vary by state, district, and even schools within the same district (Butler, 2019). Oversight is critical; for example, the CRDC (2014) concludes that Black boys represent only 8% of all students, but 18% of all students who are restrained or secluded. Since 2019, there has been increased discussion and reform regarding restraint and seclusion following numerous incidents of trauma, injury, and death. Despite media and policy attention, as well as reports from many advocacy agencies denouncing these practices, there remains a general lack of regulations and legislation that provide safeguards against these dangerous and ineffective practices (Butler, 2019).

Restraint and seclusion remain inconsistently regulated across states and districts, despite growing awareness of their harmful effects (Graves, 2023; Kern et al., 2024). State-level legislation varies widely in definitions, reporting requirements, training, and permitted forms of restraint, leaving disabled students with uneven protections (Kern et al., 2024). Scholars also argue that seclusion lacks empirical justification and should be eliminated from schools (Krezmien and Mulcahy, 2024). Interdisciplinary recommendations emphasize the importance of proactive, preventative frameworks and trauma-informed care to reduce behavioral crises that lead to restraint and seclusion (LeBel et al., 2012). These findings highlight a policy landscape marked by limited safeguards and significant variability, disproportionately impacting disabled students of color and making public attitudes toward these practices a critical area of study.

Restraint and seclusion are ineffective deterrents for misbehavior and often escalate difficult behaviors (Butler, 2019). Because of inconsistencies at the federal, state, and local levels, restraint and seclusion take many forms. However, it is important to distinguish restraint and seclusion from corporal punishment, as they are governed by different legal frameworks and justified through different institutional logics (Graves, 2023; Kern et al., 2024). This study, therefore, focuses specifically on restraint and seclusion as behavioral interventions used to immobilize or isolate students in school settings. The next section reviews nascent research on implicit ableist attitudes, which many researchers and advocates argue are at the heart of the disparate use of restraint and seclusion against disabled schoolchildren.

### Implicit ableist attitudes

The first ableist bias scale—which this current study used—was created as recently as 2019, demonstrating how new the study of implicit and explicit attitudes towards disabled people is (Friedman and Awsumb, 2019). For example, Friedman (2019) found that family members held implicit, not explicit, biases against their disabled family members and preferred the company of non-disabled persons, which demonstrates how pervasive implicit ableist attitudes are. There have been studies conducted at the school level, but they have not used a validated scale. For example, another study on teachers found that they were prejudiced against or indifferent toward students with a wide range of disabilities (Cook, 2001). One of the few studies that investigated co-constructs of race and disability found that, despite teachers’ well-meaning intentions, their efforts to combat notions of white normativity were largely ineffective (Beneke, 2021). Overall, students with disabilities are surrounded by those who hold implicit biases (healthcare providers, family members, teachers, and students) against them, which leads to deleterious consequences (inferior care, loneliness, and neglect). Though this phenomenon is well-documented, the extent to which these consequences affect the use of restraint and seclusion has yet to be explored. Taken together, existing work demonstrates widespread implicit biases against disabled people, yet research has not examined how these ableist attitudes translate into public evaluations of school discipline involving restraint and seclusion.

### Public opinion and public policy

Evidence suggests that public opinion greatly influences public policy in a myriad of ways across various domains (Burstein, 2003; Chow and Levin, 2024; Mancini et al., 2023). For example, Branham et al. (2017) found that the lower, middle, and richer classes all influence public policy to varying degrees. They contend that no one party has absolute control, but that at differing times, public policy reflects each group’s preferences. Likewise, Enns (2015)found that, despite preference gaps in the policy agenda between the richer and middle classes, both classes influence policy to a certain extent, despite their divergence. Lastly, Page and Gilens (2020)contends that the best way to combat inequity in America and the systems that disenfranchise the marginalized is through greater democracy—conceptualized as providing the public with more opportunities to influence government. Taken together, while differing members of the public influence policy across domains, it is critical that we leverage public opinion more effectively to create more equitable outcomes across government systems. This study does just that by providing readers with important information on public opinion regarding restraint and seclusion in American schools.

### Theoretical framework: DisCrit

This section reviews the seminal contributions of Annamma, Connor, and Ferri in creating Disability Critical Race Theory in 2013. This relatively new theory has shaped much of how scholars think about disability, race, and intersectionality in education. This shift in discourse was inspired by Disability Studies (Pfeiffer, 2002), Critical Race Theory (Bell, 1980; West et al., 1995), and Critical Race Theory in Education (Annamma, 2016; Ladson-Billings and Tate IV, 1995). Their work on how oppression shapes the academic and life course of disabled students of color led them to develop DisCrit (Annamma et al., 2013). Their investigation into the marginalization of disabled students of color in education showcases how ableism and racism, among other hierarchies of oppression, negatively impact the lives of these vulnerable populations. The seven key tenets are listed in Appendix 1.

Among other salient issues, DisCrit scholars focus on the overrepresentation of disabled students of color in special education (Annamma et al., 2013), the opportunity gap (Mendoza et al., 2016; Thorius and Tan, 2016), and the school-to-prison nexus (Annamma, 2014; Annamma et al., 2020). This current study highlights the school-to-prison nexus, the complex relationship all systems of governance—especially the U.S. justice system and education system—have in funneling marginalized students (such as Black students with disabilities) from the classroom to the prison cell (Sussman, 2011). The school-to-prison nexus—as opposed to the school-to-prison pipeline—recognizes the systematic, rather than individual, interest stakeholders and agencies have in embroiling students in the Prison Industrial Complex.

Drawing on the relevant DisCrit literature, the current study highlights the roles that school administrators and sworn police officers play in broad discretionary discipline policies for minor offenses, such as throwing a tantrum at school (Mahon-Reynolds and Parker, 2016). These policies, which often lead to criminal charges or fines for vulnerable youth—as early as preschool—most negatively impact disabled, American Indian, Black, and Latin* American students compared to disabled White students (Bell, 2016; Fedders, 2018). As such, this current study highlights public perceptions of American Indian and Black youth who, according to research, are most subject to discipline disparities (Fedders, 2018). Civil rights for special education populations extend mostly to White students to the exclusion of students of color (Haag, 2021; Mahon-Reynolds and Parker, 2016), so this current study explores the intersecting impact of anti-Black and anti-Indigenous biases.

DisCrit guided every aspect of the study. For example, the experimental design focused on the intersecting identities of gender, race, and disability by randomly assigning participants to six conditions: American Indian disabled, American Indian non-disabled, Black non-disabled, Black disabled, White non-disabled, and White disabled for cisgender boys. In doing so, the study captured public perceptions of these populations, which is critical to drawing attention to their plight. Secondly, as the first author, I am a disabled Black Puerto Rican with Indigenous ancestry; this study privileges marginalized voices and perspectives, which is pivotal to changing a narrative that centers on White and Western cultural norms. As the second author, I am an international scholar whose work is deeply grounded in equity-oriented research and informed by my own experiences navigating educational systems as a non-U.S. citizen and multilingual researcher. This study is motivated by a commitment to elevating marginalized perspectives and challenging narratives that privilege White, Western, and normative frameworks in education. Moreover, this study considers the legal and historical aspects of disability and race by highlighting policies regarding restraint and seclusion and capturing public perceptions of both. Restraint and seclusion are important topics for disability scholars to explore because they shape the academic and life outcomes of disabled students more so than those of non-disabled students—often with deleterious consequences (Butler, 2019). Lastly, this study—which highlights the precarious position disabled students of color hold—is a form of resistance (tenet 7) in that the article draws attention to and expresses concern for the oppression of disabled students of color.

Drawing on the literature above and guided by DisCrit, this study examines how the public evaluates restraint and seclusion when the students’ racial and disability identities vary. Specifically, the study investigates how school personnel performance, perceptions of deserved punishment, evaluations of whether the punishment fits the crime, and perceptions of prejudice shift across experimental manipulations of race and disability in an online survey experiment. In doing so, the study highlights how ableism and racism intersect to shape judgments about school discipline.

## Methodology

The research questions for the study are as follows:

1. What is the effect of the public’s implicit disability and racial biases on school personnel performance ratings?
2. What is the effect of the public’s implicit disability and racial biases on whether the child deserved the punishment?
3. What is the effect of the public’s implicit disability and racial biases on whether the punishment fit the crime?
4. What is the effect of the public’s implicit disability and racial biases on whether the incident is prejudiced?
5. How does the public view restraint and seclusion in American schools?

This study uses Disability Critical Race Theory as a framework to interpret data patterns and guide findings. DisCrit highlights how racism and ableism work together in schools, influencing how behaviors are perceived and disciplined. The study’s hypotheses assume that evaluations of restraint and seclusion are shaped not only by disability but also by racialized perceptions of disability. The experimental design tests whether public judgments differ when identical behaviors are attributed to students of varying racial and disability statuses. DisCrit emphasizes that seemingly neutral judgments often reflect normative assumptions about who is considered “typical,” “threatening,” or “in need of discipline.” Consequently, the study interprets observed differences as evidence of how social hierarchies and historical power dynamics influence public perceptions of restraint and seclusion in schools, rather than viewing race and disability solely as demographic descriptors.

This experimental study had participants observe and rate school personnel’s performance for a hypothetical scenario based on real events, as well as the degree to which the student deserved the punishment and whether the punishment fit the crime. Participants were then asked to rate the degree to which they agreed or disagreed that the events were prejudiced against the student.

Participants were randomly assigned to one of six experimental conditions that alternated the identity of the student as White non-disabled, White disabled, Black non-disabled, Black disabled, American Indian non-disabled, and American Indian disabled, based on the student’s name and mention of a special education teacher. If participant bias did not exist, then we would expect to see no statistically significant differences in personnel performance, deserved punishment or punishment fit the crime ratings, or agreement that the incident was prejudice—across these experimental conditions (either race or disability status). Because participants were randomly assigned to experimental conditions, the study is able to estimate causal effects of race and disability cues on evaluative judgments within the context of the hypothetical scenarios. However, these effects pertain specifically to responses to the vignettes and should not be interpreted as direct causal explanations of real-world behaviors.

In terms of the study’s hypotheses, it is expected that participant bias will be observed through statistically significant differences across the four outcomes, consistent with the key tenets of DisCrit. Specifically, there would be higher rates for all outcomes (1–4) when the child in question is disabled compared to non-disabled and White compared to American Indian or Black. Moreover, the four outcomes would be highest for the non-disabled White student and lowest for the disabled Black student. Per the literature, disabled American Indian students would fare better than disabled Black students, and White students would fare better than both. These hypotheses do not assume that individual respondents hold strong explicit or implicit prejudices. Consistent with DisCrit, the study examines whether racialized and ableist patterns of interpretation emerge in evaluative judgments, even among respondents who may not self-identify as biased or present as biased.

Each hypothesis reflected a key tenet of DisCrit. For example, tenet one holds that ableism and racism work in tandem, not independently, but interdependently, underscoring standards of “normal behavior.” As it relates to this experiment, results would differ depending on how disabled populations were treated versus non-disabled populations. It is hypothesized that participants would be more likely to judge the disabled student harshly because they violated notions of normalcy, especially along racial lines. Disabled White students would be treated with more compassion than disabled Black or disabled American Indian students because of the negative and compounding effects of being a disabled student and a student of color. Moreover, per tenet 3, there would be material and psychological consequences to being labeled as a student of color and/or a disabled student, such that participants’ anti-Black, anti-Indigenous, and anti-disabled biases would surface, resulting in disproportionalities across outcomes. Lastly, per tenet 6—which states that disability and Whiteness belong to White people to the exclusion of disabled people of color—it is expected that participants will treat White students and disabled White students better than American Indian students, Black students, disabled American Indian students, and disabled Black students. Having reviewed the study’s hypotheses, the data collection methods will be discussed.

### Data collection

After receiving Institutional Review Board permission (status: Exempt), data were collected via Prime Panels, an aggregate survey platform that allows researchers to examine diverse populations throughout the US quickly (Cheung et al., 2017). Prime Panels was selected because online probability-like panels have been shown to yield samples that are broader and more heterogeneous than convenience sources such as MTurk or student pools, making them useful for experimental public-opinion studies. The survey was launched and completed on February 20, 2024. The expected time to complete the survey was 6 min. The average time was 10 min and 32 s, with a median of 9 min and 45 s. After Okonofua and Eberhardt (2015) as well as Jarvis and Okonofua (2020), this experimental survey was analyzed using mixed-effects analysis of covariance (ANCOVA).

Although Prime Panels captures respondents from multiple regions of the U.S., the sample was predominantly White and older. Approximately 80% of the sample identified as White, and nearly half were aged 55–74. As such, the sample may not fully represent the broader demographic distribution of the U.S., although it certainly reflects American teachers’ race and age (USAFacts Team, 2025). These characteristics should be taken into consideration when interpreting the findings, particularly with respect to claims regarding national public opinion. Of the 1,093 surveys initiated, 770 were completed (a completion rate of approximately 70%), with incomplete responses excluded from analysis. Participants accessed the survey online through a secure Qualtrics link and were screened to confirm U.S. residence. Their state of residence was self-reported and categorized into regions based on the U.S. Census Bureau classification (Northeast, Midwest, South, West).

The study included variables such as race/ethnicity, political identity, and geographic region because these social and ideological factors are known to shape public attitudes toward school discipline and educational equity, providing important context for evaluating the vignettes. Although demographic variables such as political affiliation, region, income, and education were collected and are reported in Table 1, these variables were not included as moderators in the primary analyses. The analytic strategy was guided by the theoretical focus of the study, which centered on experimentally manipulating race and disability status to assess their influence on evaluations of restraint and seclusion. Including multiple interaction terms for all demographic factors would have substantially increased model complexity and reduced interpretability. Therefore, these variables are described descriptively, and their potential moderating role is discussed as an avenue for future research (Gelman and Hill, 2007).

**Table 1:** Means and standard deviations of continuous study variables.

Variables	*N*	*M*	*SD*	*Min*	*Max*
Bayesian Racism Scale	770	15.33	5.73	6	36
Symbolic Ableism Scale	770	3.08	0.418	1.77	4.85

The study did not ask participants about their time spent with disabled or Black individuals, as such questions often measure perceived contact rather than actual experience and are subject to social desirability bias (Greenwald and Krieger, 2006). Although contextual factors such as occupation or proximity to disabled individuals might influence disciplinary interpretations, collecting extensive contextual data could introduce complexity and noise without advancing the primary aim of manipulating identity cues experimentally (Okonofua and Eberhardt, 2015). Consequently, the analysis focused on the causal effect of experimentally manipulated race and disability status, using demographic variables only descriptively to characterize the sample.

### Scales

The current study leverages two scales: the Symbolic Ableism Scale (SAS) (Friedman and Awsumb, 2019) and the Bayesian Racism Scale (BRS) (Uhlmann et al., 2010). The SAS uncovers subtle prejudice against disabled populations. The SAS is a tool to measure ableist attitudes in order to develop strategies to counteract them (Friedman and Awsumb, 2019). The SAS ranged from a minimum of 1.77 to a maximum of 4.85, with higher values indicating greater levels of ableist beliefs. The mean of the sample for this variable was 3.08 (SD = 0.42). The BRS examines “rational racism” by using logical reasoning that supports race-based prejudice (Uhlmann et al., 2010). The BRS ranged from a minimum of 6 to a maximum of 36, with higher values indicating greater levels of racist beliefs. The mean of the sample for this variable was 15.33 (SD = 5.73). The sample descriptives for all continuous study variables are shown in Table 1.

Although neither the Symbolic Ableism Scale nor the Bayesian Racism Scale includes standardized threshold categories, both can be interpreted relative to their possible score ranges. The SAS ranges from 1.77 to 4.85, with a midpoint of approximately 3.31. The sample mean of 3.08 (SD = 0.42) therefore reflects a moderate level of symbolic ableism, somewhat below the theoretical midpoint but meaningfully above the lowest possible score. In contrast, the BRS ranges from 6 to 36, with a midpoint of 21, and the sample mean of 15.33 (SD = 5.73) falls well below that midpoint, indicating relatively low endorsement of “rational racism” beliefs in this sample. Importantly, because both scales measure broad attitudinal orientations rather than situational judgments, participants may still express compassion or concern for disabled students in specific scenarios even when general symbolic attitudes trend toward moderate bias.

The SAS and BRS were included because they measure implicit attitudinal orientations that have been shown to shape judgments in educational and disciplinary contexts (Friedman and Awsumb, 2019; Uhlmann et al., 2010). Both scales have demonstrated internal consistency reliability, predictive validity, and prior use in studies examining bias in evaluative decision-making. In this study, the scales were not used to diagnose prejudice in individuals but to contextualize how general orientations toward disability and race may relate to judgments of restraint and seclusion. Their inclusion aligns with DisCrit’s assertion that racism and ableism are embedded in evaluative systems, and the scales provide a theoretically consistent means of describing attitudinal positioning within the analytic sample.

## Results

### Description of the sample

The sample descriptives for all categorical study variables are shown in tabular form in Appendix Table A1. The analytic sample consisted primarily of women (61.6%) compared to men (38.1%). In terms of race/ethnicity, the sample was predominantly White or European (79.9%), followed by Black/African American (10.5%). The largest age group in the sample was 55–74 years of age (46.9%), followed by the 75 and above age group (16.8%). Relative to national benchmarks, the sample included a higher share of White, non-Hispanic respondents and older adults. For context, the non-Hispanic White population comprised about 58% of the U.S. population in 2023 (U.S. Census Bureau, 2024), while the national median age was approximately 39 in 2024 (U.S. Census Bureau, 2025). These differences in race and age composition may shape the public-opinion patterns observed here; accordingly, we interpret estimates as evidence of how this public sample evaluates restraint and seclusion, rather than as population-wide prevalence.

The most prevalent group in the study sample for the highest level of education received was some high school/high school degree or equivalent (25.3%), followed by some college but no degree (26.1%) and an associate’s degree (26.1%). The group with the highest proportion of household income was the less than $49,999 range (47.4%), followed by the $50,000 to $74,999 income range (26.4%). The largest concentration of participants by region of the U.S. was in the South (33.5%), followed by the Northeast (23.1%). The largest proportion of respondents identified as Democrats (35.1%), followed by Republicans (33.4%). The largest political identity group was the politically neutral group (27.1%), followed by the moderately conservative group (25.2%). Most of the sample identified as non-disabled (81.4%). The largest group randomly assigned to the experimental condition was American Indian Special Education students (17.9%), but they were generally equally distributed across the entire sample.

### Rationale for analytic approach

Given the experimental design, a mixed-effects Analysis of Covariance (ANCOVA) was used to estimate the causal effects of the race and disability conditions on evaluative outcomes while accounting for repeated measures across scenarios. This approach is well established in school discipline vignette experiments in which participants evaluate multiple related events that share context but differ systematically in escalation or personnel involvement (Okonofua and Eberhardt, 2015; Jarvis and Okonofua, 2020). The mixed-effects structure allows the model to account for within-participant correlation across scenarios while examining between-participant variation attributable to the randomized identity cues. Prior to analysis, assumptions of ANCOVA (linearity, normality of residuals, homogeneity of regression slopes, and independence of errors) were tested. No violations requiring model adjustment were detected.

### Experimental condition structure

Participants were randomly assigned to one of six race/disability identity conditions. Random assignment supports internal validity by ensuring that any observed differences in evaluations are causally attributable to the identity cues rather than participant characteristics. Participants evaluated multiple scenarios involving different personnel (teacher, principal, school police officer), but the identity condition remained constant for each respondent (Mutz, 2011). This structure prevents respondents from detecting the experimental manipulation and reduces demand characteristics (Orne, 1962). It also reflects the DisCrit-informed logic that identity is socially interpreted as stable and backgrounded when evaluating behavior (Annamma et al., 2013). Although DisCrit emphasizes the interdependence of race and disability, the factorial design allows the authors to test whether identity cues influenced evaluations without assuming independence of these social constructs; interactions were examined and are reported.

### Balance tests

To test the effectiveness of the randomization of the participants into experimental groups, balance tests were conducted to ensure that there were no significant participant differences across experimental conditions (Gerber and Green, 2012). Categorical demographic factors and the continuous variables were analyzed with the experimental groups using crosstabulations (see Appendix Table 2) and a one-way ANOVA (see Table 2).

**Table 2:** Means and standard deviations of the Bayesian Racism Scale and Symbolic Ableism Scale by experimental conditions.

Bayesian Racism Scale	*N*	*M*	*SD*	*F*	*p*
Experimental condition				1.19	0.313
White	124	14.57	5.81		
White disabled	127	14.78	5.58		
Black	131	15.27	5.79		
Black disabled	120	15.53	5.62		
Native American	130	15.86	6.02		
Native American disabled	138	15.89	5.54		

Symbolic Ableism Scale	*N*	*M*	*SD*	*F*	*p*
Experimental condition				1.43	0.212
White	124	3.08	0.41		
White disabled	127	3.10	0.40		
Black	131	3.00	0.38		
Black disabled	120	3.09	0.43		
Native American	130	3.13	0.50		
Native American disabled	138	3.10	0.38		

The results indicate that most of these factors were not statistically significant in these tests, *p* > 0.05. However, one factor, age, was statistically significant in these balance tests. There were significantly more participants in the 55 to 74 age group assigned to the Black non-disabled condition (55.7%) compared to the Black disabled condition (35.0%), *ps* < 0.05. Accordingly, the age variable was included in all further analyses to control for this imbalance across the experimental conditions.

### Outcome 1: personnel performance outcome

The personnel performance outcome analysis was conducted using a mixed effects analysis of covariance (ANCOVA), which controls for both repeated measures variation (scenarios) and between-subjects variation (experimental conditions and other covariates) (Gelman and Hill, 2007). The outcome was measured on a five-point Likert-type scale that captured Failing, Poor, Mediocre, Good, and Excellent performance, with higher levels indicating better performance. The independent variables for this study consisted of the experimental conditions represented as two dichotomous variables, Experimental condition-Race (Exp. Race) and Experimental condition-Disability (Exp. Disability), to examine race and disability biases separately, as well as the interaction, and the scenario (teacher restraint, principal suspension, officer called, officer restraint). Age was included as a control variable. Effect sizes for the main effects in the model are represented using partial eta-squared (*η*^2^*_p_*).

The results of the mixed effects ANCOVA are shown in Table 3. The model results indicate that the main effects of Disability (*η*^2^*_p_* = 0.015, *p* = 0.001), scenario (*η*^2^*_p_* = 0.008, p = 0.001), and age (*η^2^_p_* = 0.01, *p* = 0.005) were all statistically significant in the model. For the experimental condition-disability status, school personnel were rated higher on their performance for non-disabled students (M = 3.56, SE = 0.05) compared to disabled students (M = 3.34, SE = 0.05), *p* < 0.001. For scenario, there was a de-escalatory effect with ratings decreasing (performance rated worse) as the scenarios increased in intensity. School personnel were rated higher on their performance for the teacher restraint scenario (M = 3.77, SE = 0.04) and the principal suspension scenario (M = 3.66, SE = 0.04) compared to the officer called scenario (M = 3.31, SE = 0.05) and the officer restraint scenario (M = 3.06, SE = 0.05), *ps* < 0.001. All estimated marginal means were evaluated holding the ordinal age variable at its mean (M = 4.43).

**Table 3:** Mixed effects ANOVA testing performance ratings of school personnel by scenario and experimental condition, controlling for age.

Source	*SS*	*df*	*MS*	*F*	*p*	*η*^2^
Between-subjects effects						
Intercept	2365.17	1	2365.17	720.39	0.001	0.486
Age	26.21	1	26.21	7.98	0.005	0.010
Exp. Race	9.79	2	4.89	1.49	0.226	0.004
Exp. Disability	38.88	1	38.88	11.84	0.001	0.015
Exp. Race*Exp. Disability	0.24	2	0.12	0.04	0.963	0.000
Error	2505.08	763	3.28			
Within-subjects effects						
Scenario	14.49	2.63	5.51	5.97	0.001	0.008

SS is the sum of squares. MS is mean squares, *η*^2^*_p_* is the effect size or partial eta-squared.

Appendix Figures 1, 2 provide the estimated marginal mean ratings of personnel for each of the experimental conditions within each hypothetical scenario while controlling for the covariates in the model. For the teacher restraint scenario, the disabled (*M* = 3.76, *SE* = 0.05) and non-disabled (*M* = 3.78, *SE* = 0.05) groups were rated very similarly; however, in the three subsequent scenarios, the performance in reference to a non-disabled student was higher than that of a disabled student. Additionally, Appendix Figure 1 shows the lack of differences in performance ratings by student race in experimental conditions.

### Outcome 2: deserved punishment outcome

The deserved punishment outcome analysis was conducted using a mixed-effects analysis of covariance (ANCOVA), which controls for both repeated measures variation (scenarios) and between-subjects variation (experimental conditions and other covariates). The outcome was measured using a six-point Likert-type scale ranging from Strongly disagree to Strongly agree, with higher levels indicating stronger agreement that the student deserved the punishment. The independent variables for this study consisted of the experimental conditions represented as two dichotomous variables (Exp. Race and Exp. Disability) to examine race and disability biases separately as well as the interaction, and the scenario (principal suspension, officer restraint). Age was included as a control variable. Effect sizes for the main effects in the model are represented using partial eta-squared (*η*^2^*_p_*).

Table 4 presents the results of the mixed-effects ANCOVA. The model results indicate that the main effects of Disability (*η*^2^*_p_* = 0.038, *p* < 0.001), scenario (*η*^2^*_p_* = 0.039, *p* < 0.001), and age (*η*^2^*_p_* = 0.008, *p* = 0.012) were all statistically significant in the model. For the experimental condition-disability status, the student was rated higher than they deserved the punishment for non-disabled students (*M* = 4.45, *SE* = 0.07) compared to disabled students (*M* = 3.93, *SE* = 0.07), *p* < 0.001. For the scenario, on average, all students were rated higher than they deserved the punishment for principal suspension (*M* = 4.58, *SE* = 0.05) more than for officer restraint/handcuffing (*M* = 3.80, *SE* = 0.06), *p* < 0.001. All estimated marginal means were evaluated holding the ordinal age variable at its mean (*M* = 4.43).

**Table 4:** Mixed effects ANOVA testing deserve punishment ratings by scenario, experimental condition controlling for age.

Source	*SS*	*df*	*MS*	*F*	*p*	*η^2^*
Between-subjects effects						
Intercept	1728.39	1	1728.39	509.26	0.001	0.400
Age	21.58	1	21.58	6.36	0.012	0.008
Exp. Race	3.23	2	1.61	0.48	0.622	0.001
Exp. Disability	102.46	1	102.46	30.19	0.001	0.038
Exp. Race*Exp. Disability	0.36	2	0.18	0.05	0.949	0.000
Error	2589.58	763	3.39			
Within-subjects effects						
Scenario	33.14	1.00	33.14	31.12	0.001	0.039

SS is the sum of squares. MS is mean squares, *η*^2^*_p_* is the effect size or partial eta-squared.

Appendix Figures A3, A4 provide the estimated marginal mean ratings of personnel for each of the experimental conditions within each hypothetical scenario while controlling for the covariates in the model. For the principal suspend scenario, the non-disabled student (*M* = 4.87, *SE* = 0.07) was rated much higher as deserving the punishment than the disabled student (*M* = 4.30, *SE* = 0.07). For the officer restrain scenario, the same trend is apparent as the non-disabled student (*M* = 4.04, *SE* = 0.08) was rated much higher as deserving the punishment than the disabled student (*M* = 3.57, *SE* = 0.08). Additionally, the visual in Appendix Figure A3 shows the lack of differences in performance ratings by student race categories. The same trend can be observed across both visuals, with all students rated as deserving the principal suspension more than the officer restrain/handcuff punishment.

### Outcome 3: punishment fits the crime outcome

The punishment fits the crime outcome analysis was conducted using a mixed effects analysis of covariance (ANCOVA). The outcome was measured using a six-point Likert-type scale that captured the degree to which participants believed that the student’s punishment fit their crime, with higher values indicating greater agreement that the punishment fit the crime. The independent variables for this study consisted of the experimental conditions represented as two dichotomous variables (Exp. Race and Exp. Disability) to examine race and disability biases separately, as well as the interaction, and the scenario (principal suspend, officer restraint). Age was included as a control variable. Effect sizes for the main effects in the model are represented using partial eta-squared (*η*^2^*_p_*).

The results of the mixed effects ANCOVA are shown in Table 5. The model results indicate that the main effects of Disability (*η*^2^*_p_* = 0.017, *p* < 0.001) and scenario (*η*^2^*_p_* = 0.032, *p* < 0.001) were statistically significant in the model. For the experimental condition—disability status, the non-disabled students (*M* = 4.12, *SE* = 0.07) were rated higher for the punishment fitting the crime compared to disabled students (*M* = 3.76, *SE* = 0.07), *p* < 0.001. For the scenario, students were rated higher that the punishment fit the crime for the principal suspension (*M* = 4.24, *SE* = 0.05) than the officer restrain/handcuff (*M* = 3.65, *SE* = 0.06), *p* < 0.001. All estimated marginal means were evaluated holding the ordinal age variable at its mean (*M* = 4.43).

**Table 5:** Mixed effects ANOVA testing punishment fit the crime ratings by scenario and experimental condition, controlling for age.

Source	*SS*	*df*	*MS*	*F*	*p*	*η*^2^
Between-subjects effects						
Intercept	1578.08	1	1578.08	430.81	0.001	0.361
Age	13.75	1	13.75	3.75	0.053	0.005
Exp. Race	8.80	2	4.40	1.20	0.301	0.003
Exp. Disability	49.53	1	49.53	13.52	0.001	0.017
Exp. Race*Exp. Disability	4.18	2	2.09	0.57	0.566	0.001
Error	2794.92	763	3.66			
Within-subjects effects						
Scenario	26.67	1.00	26.67	25.08	0.001	0.032

SS is the sum of squares. MS is mean squares, *η*^2^*_p_* is the effect size or partial eta squared.

Appendix Figures 5, 6 provide the estimated marginal means of punishment fit the crime ratings for the experimental conditions within each scenario while controlling for the covariates in the model. For principal suspension, the non-disabled student group (*M* = 4.42, *SE* = 0.07) was higher compared to the disabled student group (*M* = 4.06, *SE* = 0.07), indicating higher agreement that the punishment fit the crime for non-disabled students than for disabled students. In the officer restrain/handcuff scenario, the non-disabled student group (*M* = 3.83, *SE* = 0.08) was higher compared to the disabled student group (*M* = 3.47, *SE* = 0.08), again indicating higher agreement that the punishment fit the crime for non-disabled students than for disabled students. Additionally, Appendix Figure 4 shows that there were minor, yet not statistically significant, differences in punishment fit the crime ratings between racial experimental groups within scenarios. The visuals demonstrate that across both race and disability experimental conditions, the ratings indicated higher agreement that the punishment fit the crime for the principal suspension than for the officer restrain/handcuff scenario.

### Outcome 4: prejudice outcome

The prejudice outcome analysis was conducted using an analysis of covariance (ANCOVA) with no within-subjects modeling, as this question was not asked for each scenario repeated measure. The outcome was measured on a six-point Likert-type scale measuring levels of disagreement to agreement that the treatment of the student was an incident of prejudice, with higher values indicating greater agreement that it was prejudice. The independent variables for this study consisted of the experimental conditions represented as two dichotomous variables (Exp. Race and Exp. Disability) to examine race and disability biases separately, as well as their interaction. Age was included as a control variable. Effect sizes for the main effects in the model are represented using partial eta-squared (*η*^2^*_p_*).

The results of this prejudice model are shown in Table 6. The model results indicate that only the effect of age (*η*^2^*_p_* = 0.031, *p* < 0.001) was statistically significant. Although not significantly higher, participants rated higher levels of prejudice for disabled students (*M* = 2.70, *SE* = 0.07) compared to non-disabled students (*M* = 2.53, *SE* = 0.07), *p* = 0.09. As a pairwise comparison, participants rated significantly higher levels of prejudice for Black students (*M* = 2.74, *SE* = 0.09) than for White students (*M* = 2.49, *SE* = 0.09), *p* < 0.05. No other racial pairwise comparisons were significantly different from one another. Appendix Figures 7, 8 provide the estimated marginal mean ratings of prejudice for each of these experimental conditions while controlling for the covariates in the model. All estimated marginal means were evaluated holding the ordinal age variable at its mean (*M* = 4.43).

**Table 6:** Mixed effects ANOVA testing prejudice by experimental condition controlling for age.

Source	*SS*	*df*	*MS*	*F*	*p*	*η*^2^
Corrected model	67.04	6	11.17	5.74	0.001	0.043
Intercept	726.22	1	726.22	373.21	0.001	0.328
Age	47.60	1	47.60	24.46	0.001	0.031
Exp. Race	8.33	2	4.16	2.14	0.118	0.006
Exp. Disability	5.63	1	5.63	2.90	0.089	0.004
Exp. Race*Exp. Disability	2.05	2	1.02	0.53	0.591	0.001
Error	1484.71	763	1.95			

SS is the sum of squares. MS is mean squares, *η*^2^*_p_* is the effect size or partial eta squared.

### Public perceptions of restraint and seclusion and word cloud

Among other advancements in theory, research, and practice, this study makes an important policy contribution by capturing—for the first time—public opinion on restraint and seclusion in U.S. schools. In terms of public approval for restraint and seclusion, participants were more likely to support the use of restraint and seclusion but agreed that there should be clear state laws on their use in U.S. schools (see Table 7 for a breakdown).

**Table 7:** Public opinions on restraint and seclusion.

Response	N	%
There should be clear state laws on the use of restraint in schools in the US?		
Strongly agree	290	37.7
Somewhat agree	151	19.6
Agree	269	34.9
Disagree	12	1.6
Somewhat disagree	35	4.5
Strongly disagree	12	1.6
Restraint should never be used in US schools.		
Strongly agree	47	6.1
Somewhat agree	109	14.2
Agree	62	8.1
Disagree	174	22.6
Somewhat disagree	249	32.3
Strongly disagree	127	16.5
There should be clear state laws on the use of seclusion in schools in the US?		
Strongly agree	221	28.7
Somewhat agree	207	26.9
Agree	234	30.4
Disagree	24	3.1
Somewhat disagree	60	7.8
Strongly disagree	24	3.1
Seclusion should never be used in US schools.		
Strongly agree	73	9.5
Somewhat agree	146	19
Agree	98	12.7
Disagree	127	16.5
Somewhat disagree	241	31.3
Strongly disagree	84	10.9

### Summary of results

These results indicate that for the rating of the school personnel’s performance *(Outcome 1),* the performance rating of personnel disciplining the hypothetical student was significantly higher for the non-disabled student group compared to the disabled student group, controlling for scenario type and other covariates; there were no statistically significant differences across race groups within scenarios. This suggests that participants thought punishing non-disabled students was more acceptable than punishing disabled students. For the rating of whether the punishment was deserved *(Outcome 2),* the same trend was observed (including no differences across racial experimental conditions within scenarios). This suggests that to the average participant, a student deserved their punishment more if they were not disabled compared to the disabled hypothetical student. For the rating of whether the punishment fit the crime *(Outcome 3),* again the same trends were observed. This suggests that, on average, a student’s punishment fit the crime more if the student was not disabled compared to the hypothetical disabled student. Lastly, the participants were asked if they thought the incidents were instances of prejudice *(Outcome 4),* irrespective of the kind of scenario.

The participants, on average, indicated that the incident being prejudicial was significantly higher for the Black student condition compared to the White student condition, while controlling for covariates; there were no significant pairwise differences between White and American Indian students or Black and American Indian students. Additionally, there were no significant differences on this variable for the disability experimental condition, though on average, prejudice was rated higher for the disabled student group than for the non-disabled students. This suggests that, for the average participant, Black American students received more sympathy with regard to prejudice compared to the White students in the experiment.

## Discussion

In this article, a randomized survey experiment was used to explore four questions about how the public evaluates the use of restraint and seclusion in schools for student groups that varied by race (American Indian, Black, or White) and disability (disabled or non-disabled). The research question was: what is the effect of the public’s implicit disability and racial biases on (1) school personnel performance ratings, (2) whether the child deserved the punishment, (3) whether the punishment fit the crime, and lastly, (4) whether the incident was prejudiced. Additionally, the study was interested in public opinions on restraint and seclusion. This study hypothesized that DisCrit would result in the four outcomes being statistically different for the disabled student compared to the non-disabled student, which reflects larger trends in the literature (Annamma, 2014; Annamma, 2016). Furthermore, it is hypothesized that the public would treat disabled Black students, then disabled American Indian students, more harshly than disabled White students—based on prior literature that suggests that disabled Black students have the highest incidences of restraint and seclusion, followed by disabled American Indians (Butler, 2019; Restraint and Seclusion Data, 2020). Instead, the study participants held greater sympathy for the disabled student than for the non-disabled student, and there were no statistically significant differences across racial groups.

One explanation for the absence of expected racialized differences may relate to the nature of the experimental task. Because the vignettes were presented in an online and hypothetical context, participants were not situated within the embodied, relational, and institutional conditions under which racialization typically occurs in school settings. Race cues in written scenarios may be less salient than in face-to-face interactions, particularly when the behavior is pre-scripted and the student’s affect cannot be perceived (Okonofua and Eberhardt, 2015). Additionally, social desirability pressures may have influenced responses, especially given that the sample skewed older and racial discourse has become a highly visible public issue (Krumpal, 2013). In this context, expressing race-neutral judgments may reflect awareness of racial sensitivity rather than the absence of racialized meaning-making. From a DisCrit perspective, the lack of observed racial differentiation should not be interpreted as evidence of racial equity, but as part of the systemic invisibility through which ableism and anti-Blackness operate. Racialized disability is often rendered normative, taken for granted, and therefore unnoticed in evaluative judgments. The apparent uniform sympathy toward disabled students in this study may thus align with DisCrit’s assertion that racism and ableism function most powerfully when they are unseen rather than explicit.

These findings can be understood in relation to prior research showing that implicit bias may emerge not only from personal attitudes but also from institutional and professional norms surrounding disability (Cook, 2001; Friedman and Awsumb, 2019). This study did not directly assess participants’ personal or professional contact with disabled individuals; therefore, no conclusions can be drawn about the relationship between contact and bias. Instead, the observed pattern that disabled students were evaluated more sympathetically may reflect the broader cultural framing of disability as associated with vulnerability, care, or reduced responsibility (Garland-Thomson, 2020). From a DisCrit perspective, this does not necessarily indicate equitable treatment; rather, it may demonstrate how disability is positioned in public imagination in ways that elicit pity or compassion while still reinforcing categories of normalcy and difference. Thus, the results highlight how the social meaning attached to disability can shape evaluative judgments, even when explicit bias is low or unmeasured.

### Policy and practice recommendations

Research shows that restraint and seclusion in schools can be significantly reduced or even eliminated when schools adopt proactive behavior support frameworks and trauma-informed practices (Graves, 2023; LeBel et al., 2012). Approaches like Positive Behavioral Interventions and Supports (PBIS) and restorative practices focus on de-escalation, predictable routines, and collaborative problem-solving, which help prevent the behavioral crises that often lead to restraint and seclusion (Krezmien and Mulcahy, 2024). However, inconsistent policy language and enforcement across states exacerbate the disproportionate use of these practices, particularly for racially minoritized disabled students (Kern et al., 2024). To address this, clear federal and state guidelines, combined with professional development for educators and school resource officers, are essential for reducing harmful exclusionary practices.

Schools are increasingly adopting training programs to reduce implicit racial and ableist biases among educators and staff, recognizing that without targeted professional learning, staff may perceive the behaviors of racially minoritized disabled students as more intentional or threatening than those of their non-disabled or White peers (Cook, 2001; Annamma et al., 2020). Many states now require districts to monitor discipline data for disproportionality and implement corrective action plans when disparities exceed state thresholds (Kern et al., 2024).

Although federal monitoring through the Civil Rights Data Collection remains active, the second Trump administration has signaled shifts away from equity-focused discipline guidance and encouraged stricter disciplinary regimes (Williams, 2025). These policy reversals pose a danger of regression for students, especially racially minoritized and disabled youth, who remain disproportionately at risk for physical injury, trauma, and even death when disciplinary oversight, data monitoring, and equity-oriented training are scaled back. As federal protections weaken, it becomes increasingly important for researchers, advocates, and local educational agencies to maintain vigilance in monitoring discipline practices and safeguarding vulnerable student populations. Strategies such as implicit bias training, coaching within Positive Behavioral Interventions and Supports (PBIS) frameworks, and restorative practice training have been effective in reducing escalated responses that lead to restraint or seclusion, particularly when combined with ongoing data review and accountability systems (Graves, 2023). These findings suggest that public attitudes, especially those that view the restraint of disabled students as less justified, may support continued efforts to reform discipline practices and address disproportionality in the use of restraint and seclusion.

In regard to the average participant believing the incidence was more prejudiced when the hypothetical student was Black than when the hypothetical student was White, while recent national conversations about racial injustice have brought increased visibility to the experiences of Black and Native American communities, research consistently shows that both implicit and explicit racial biases continue to shape public interpretation of student behavior and school discipline outcomes (Annamma et al., 2020; Okonofua and Eberhardt, 2015). Therefore, the patterns observed in this study should be understood not as evidence of a shift away from bias but as part of an ongoing and uneven landscape in which race continues to influence perceptions of restraint and seclusion. While there is some support from the literature, this study should be replicated to determine if findings are due to chance. These findings indicate that the general public may hold more critical views of restraint and seclusion than have been reflected in school-based disciplinary practices. This is consistent with research showing that school personnel often operate within institutional cultures that normalize restraint and seclusion despite documented risks (LeBel et al., 2012).

The narrative that opened this study illustrates how individual incidents can catalyze public scrutiny and policy reconsideration (Mizner, 2018). This study provides empirical support that can inform ongoing legislative and school reform efforts aimed at reducing restraint and seclusion and promoting equitable, non-harmful approaches to student support. A major contribution of this current study is the evidence of a misalignment between public policy regarding restraint and seclusion in American schools and public opinion on its regulation and use. This study finds that when presented with scenarios involving restraint and seclusion, respondents tended to favor stronger limits and accountability measures for these practices. This reflects a preference for protective oversight rather than demonstrating detailed knowledge of current state or federal regulations. It begs the question of why we use restraint and seclusion if there is a general lack of support for both. One reason we use restraint and seclusion in schools despite this misalignment could be the lack of research on public opinion regarding restraint and seclusion in American schools. Now that we have new, critical information on their opinions, it is important to galvanize policy stakeholders to action. Another reason could be that the U.S. public surveys disabled bodies because they violate supposed social expectations of normality (Annamma, 2016). However, the current research contradicts this notion: perhaps teachers—who work with students daily and maintain interpersonal contact with them—hold expectations of normalcy, but the general public does not. Public opinion can contribute to shaping school discipline policy, but meaningful change requires alignment with evidence-based alternatives to restraint and seclusion and clear regulatory guidance (Kern et al., 2024).

These findings may help guide ongoing policy and professional development efforts. For example, state and district policies could more clearly restrict the use of restraint and seclusion to emergency situations involving immediate physical danger, strengthen reporting requirements, and mandate parent notification. Additionally, teacher preparation programs and school resource officer training could incorporate structured modules on disability, de-escalation, and the ways racialized perceptions of behavior shape disciplinary decision-making. At the same time, public empathy toward disabled students does not itself translate into equitable outcomes. DisCrit scholarship emphasizes that structural accountability, rather than attitudinal change alone, is necessary to disrupt the routine and institutionalized nature of racialized ableism in schools (Annamma et al., 2013). Therefore, aligning policy with public sentiment requires enforcement mechanisms, ongoing monitoring of disproportionality, and sustained professional learning that addresses how race and disability are co-constructed in school contexts.

### Limitations

A key limitation of this study is the demographic composition of the participant sample. The majority of respondents were White and older adults, groups that historically exhibit different patterns of racial and disability-related attitudes compared to younger and more racially diverse populations (Administration for Community Living, 2024). Therefore, while the findings provide insight into how members of the public evaluate restraint and seclusion, they may not fully capture the perspectives of racially minoritized communities or younger generations. However, it is relevant to note that the population of K–12 school principals in the U.S. is itself majority White (77% non-Hispanic White for public school principals in 2020–21) and older than the general teaching population, with mean ages near the late 40s (National Center for Education Statistics, 2022). This alignment suggests the sample may approximate the demographic characteristics of the officials (principals/superintendents) who are often the first decision-makers in discipline incidents, which slightly tempers the limitation regarding generalizability. Future research should replicate this experimental design with more demographically diverse samples to assess whether these patterns hold across racial, cultural, and generational contexts.

Another limitation is that demographic variables such as political orientation, region, income, and education were not analyzed as moderators of participants’ responses. Prior research indicates that political ideology and cultural context can shape attitudes toward discipline, punishment, and perceived fairness in schools (Okonofua and Eberhardt, 2015). Therefore, it is possible that subgroup differences may exist within the public that were not identified in this study. Future research should examine how these demographic factors interact with racialized and ableist perceptions to better understand variation in public attitudes toward restraint and seclusion.

It is also possible that the language used in the survey influenced participants’ interpretations of the scenarios. Although the scenarios were intentionally written in neutral, behaviorally descriptive terms to avoid emphasizing emotional tone or student blame, certain terms such as “restraint,” “suspension,” and “school police officer” may carry strong cultural associations. These associations could prompt participants to rely on pre-existing assumptions about disability, authority, and school discipline rather than forming judgments solely on the information provided. Additionally, the scenarios did not describe the student’s emotional state, intent, or prior history, which may have led respondents to fill in these gaps using cultural narratives about normativity and responsibility. From a DisCrit perspective, this is not a flaw but rather an illustration of how meaning-making around disability and race operates through shared language and institutional discourse. Nonetheless, the influence of scenario wording on evaluative judgments should be considered when interpreting the findings, and future research may experiment with varied language framing to test how linguistic cues shape public responses to restraint and seclusion.

### Policy implications

Here, multiple implications for research and practice based on the current study findings have been listed. In terms of research, more studies are needed to tease out the implications of being disabled and racially minoritized. While this study finds that disability took primacy over race, research and theoretical frameworks like DisCrit support the interdependent relationship between disability and race. As such, more bias-focused research on the interplay of race and disability and how that impacts discipline outcomes is needed. In terms of implications for DisCrit specifically, the current research supports the potentially positive role the public could play in advancing social justice for racially minoritized disabled students. DisCrit scholarship is most often applied to education, but this current research supports its application to public policy (Annamma et al., 2013). DisCrit scholars may consider leveraging public advocacy in educational issues centering on discipline disparities among racially minoritized disabled students.

Likewise, in terms of practice, schools should involve the broader public or local community in determining whether they support the restraint and seclusion of disabled students. A lack of local support for restraint and seclusion or support for more regulation regarding restraint and seclusion could positively influence discipline disparities related to restraint and seclusion, especially for racially minoritized disabled students. Butler (2019) finds that even public conversations about restraint and seclusion reduce or regulate its use in schools. This research supports the potential power of the public to shape policies regarding restraint and seclusion in American schools.

Lastly, this research has important implications for QuantCrit, as the experimental survey design on public opinion demonstrates the untapped power of aggregate survey platforms and randomized experiments. Largely, QuantCrit research does not leverage randomized experiments despite this design being strong in causal inference; instead, researchers primarily use descriptive statistical analyses and secondary data analyses (Campbell, 2020; Campbell-Montalvo, 2020). This current study evidences how experimental designs can advance equity and social justice in education. Likewise, this is one of the only QuantCrit studies that leverages an aggregate survey platform despite the fact that these platforms are more diverse than traditional U.S. samples (i.e., undergraduates) (Chandler et al., 2019; Cheung et al., 2017; Hunt and Scheetz, 2019).

Additionally, this study makes several critical contributions to knowledge. In terms of scope, the survey design—which includes American Indian student experiences—combats the repeated erasure of Indigenous populations in research (Pettigrew, 2011). Moreover, this study examines the intersection of race and disability, which advances our understanding of disabled groups of color who continually elude public and research attention and concern (Annamma et al., 2013; Beneke, 2021). Methodologically, this is the first experimental application of DisCrit, which is important to understanding anti-disability bias as it intersects with anti-Black bias and anti-Indigenous bias among the American public. Substantively, this study is part of a new wave of research on public perceptions of critical school discipline issues (Author, year; Author, year). However, this is the first experimental study to examine disability bias in school discipline procedures. Friedman (2019)—a well-known disability scholar—recently called for more studies that examine implicit and explicit ableist biases.

Additionally, this research is crucial because disabled schoolchildren represent a growing share of students (12% as of 2014) and an even larger share of schoolchildren who are secluded (58%) and restrained (75%) (CRDC, 2014). Likewise, this is the first school discipline experiment on restraint and seclusion in U.S. schools, which leads to multiple deaths of disabled children each year (Butler, 2019; Kutz, 2009). Research on restraint and seclusion has historically studied its use in psychiatric centers or hospitals, but there are only a handful of empirical studies that examine restraint and seclusion use in schools (Ryan and Peterson, 2004). Importantly, the restraint and seclusion of disabled students has increased in the U.S. (CRDC, 2014, 2019); this research is important for expanding our understanding of the intersections of disability and race in harsh school discipline procedures.

Here, the research provides directions for subsequent scholarship. First, this study focused on the disability and racial identities of cisgender boys for sufficient power and because these identities are in jeopardy, but future research should examine the intersection of other salient identities, such as those of disabled, transgender, or gender-expansive students. This research could potentially illuminate the precarious positions multiply marginalized students hold and help us better create solutions for those most in jeopardy. Second, this study surveyed the general public because their influence on policy is undeniable, and a primary purpose of this article is to improve policies regarding restraint and seclusion. Still, researchers should survey school personnel, including SLEOs and SROs, to better understand how racial and disability biases intersect to create disparities in restraint and seclusion. Lastly, this article highlighted restraint and seclusion, but more research is needed on related school discipline practices, such as corporal punishment, which is still practiced in the U.S. Overall, while this study makes important contributions to knowledge, there is room for further advancement.

## Conclusion

To close, the findings fill a critical gap in the literature related to restraint and seclusion among racially minoritized disabled students and help us understand the profound potential role the public can play in ameliorating the disproportionate use of restraint and seclusion against racially minoritized disabled students. This study also demonstrates the value of applying DisCrit to public policy research, in this case, by situating restraint and seclusion as problematic, rather than racially minoritized disabled students. Future research on the topic of restraint and seclusion in U.S. schools should deepen and extend the use of DisCrit as a theoretical framework to guide study design, research methods, and analysis. Our ability to disrupt inequity in the use of seclusion and restraint in schools is contingent on the study of public perceptions of it, as the public has great influence on policy. To create equitable outcomes in restraint and seclusion or to do away with them altogether, we must think in new ways about disability, race, bias, and research.

## Data Availability

The raw data supporting the conclusions of this article will be made available by the authors, without undue reservation.
