# Supplementary material for: Handcuffed for 15 min: public perceptions of restraint and seclusion in schools: an experimental study of race and disability
Source: Front Psychol. 2026 Jan 23;16:1646644. doi: 10.3389/fpsyg.2025.1646644 (PMC12876222; doi:10.3389/fpsyg.2025.1646644)
Supplement: Supplementary file 1 [file Supplementary_file_1.docx]

**Appendix**

This Appendix includes multiple tables and figures that support the methodology and results.

**TABLES**

**Appendix Table A1**

*Frequencies and Percentages of Categorical Study Variables*

______________________________________________________________________________

| Variables | | *n* | | *%* |
| --- | --- | --- | --- | --- |
|  |  |  |  |  |
| Gender | |  |  |  |
|  | Man | 293 |  | 38.1 |
|  | Woman | 474 |  | 61.6 |
|  | Missing | 3 |  | .4 |
| Age | |  |  |  |
|  | 18-24 years | 30 |  | 3.9 |
|  | 25-34 years | 49 |  | 6.4 |
|  | 35-44 years | 97 |  | 12.6 |
|  | 45-54 years | 104 |  | 13.5 |
|  | 55-74 years | 361 |  | 46.9 |
|  | 75 and above | 129 |  | 16.8 |
|  |  |  |  |  |
| Race/ethnicity | |  |  |  |
|  | Asian or Pacific Islander | 20 |  | 2.6 |
|  | Black or African American | 81 |  | 10.5 |
|  | Hispanic or Latina/o/x | 24 |  | 3.1 |
|  | White or European | 615 |  | 79.9 |
|  | Not listed, please specify | 30 |  | 3.9 |
|  |  |  |  |  |
| Highest level of education | |  |  |  |
|  | Some high school/ High school degree or equivalent | 195 |  | 25.3 |
|  | Some college but no degree | 201 |  | 26.1 |
|  | Associate degree | 112 |  | 26.1 |
|  | Bachelor’s degree | 168 |  | 21.8 |
|  | Graduate degree | 94 |  | 12.2 |
|  |  |  |  |  |
| Household income | |  |  |  |
|  | Less than $49,999 | 365 |  | 47.4 |
|  | $50,000 to 74,999 | 203 |  | 26.4 |
|  | $75,000 to 99,999 | 86 |  | 11.2 |
|  | $100,000 to $124,999 | 47 |  | 6.1 |
|  | $125,000 to $149,000 | 22 |  | 2.9 |
|  | $150,000 to $174,999 | 20 |  | 2.6 |
|  | $175,000 or higher | 27 |  | 3.5 |
|  |  |  |  |  |
| Region | |  |  |  |
|  | Northeast | 178 |  | 23.1 |
|  | South | 258 |  | 33.5 |
|  | Midwest | 162 |  | 21.0 |
|  | West | 172 |  | 22.3 |
|  |  |  |  |  |
| Political party | |  |  |  |
|  | Democrat | 270 |  | 35.1 |
|  | Independent | 210 |  | 27.3 |
|  | Republican | 257 |  | 33.4 |
|  | Not listed | 33 |  | 4.3 |
|  |  |  |  |  |
| Political identity | |  |  |  |
|  | Strongly conservative | 117 |  | 15.2 |
|  | Moderately conservative | 194 |  | 25.2 |
|  | Neutral | 209 |  | 27.1 |
|  | Moderately liberal | 152 |  | 19.7 |
|  | Strongly liberal | 75 |  | 9.7 |
|  | Not listed | 23 |  | 3.0 |
|  |  |  |  |  |
| Disabled | |  |  |  |
|  | No | 627 |  | 81.4 |
|  | Yes | 143 |  | 18.6 |

**Appendix Table A2**

*Frequencies and Percentages of Demographics by Experimental Conditions*

____________________________________________________________________________________________________________

|  |  | White | |  | White Disabled | |  | Black | |  | Black Disabled | |  | Native American | |  | Native American Disabled | |  |  |  |  |
| --- | --- | --- | --- | --- | --- | --- | --- | --- | --- | --- | --- | --- | --- | --- | --- | --- | --- | --- | --- | --- | --- | --- |
|  |  | *n* | *%* |  | *n* | *%* |  | *n* | *%* |  | *n* | *%* |  | *n* | *%* |  | *n* | *%* |  | *χ^2^* | *p* | |
|  |  |  |  |  |  |  |  |  |  |  |  |  |  |  |  |  |  |  |  |  |  | |
| Gender | |  |  |  |  |  |  |  |  |  |  |  |  |  |  |  |  |  |  | 1.18 | .947 | |
|  | Man | 47 | 37.9 |  | 44 | 34.6 |  | 51 | 39.8 |  | 46 | 38.3 |  | 49 | 37.7 |  | 56 | 40.6 |  |  |  | |
|  | Woman | 77 | 62.1 |  | 83 | 65.4 |  | 77 | 60.2 |  | 74 | 61.7 |  | 81 | 62.3 |  | 82 | 59.4 |  |  |  | |
|  |  |  |  |  |  |  |  |  |  |  |  |  |  |  |  |  |  |  |  |  |  | |
| Age | |  |  |  |  |  |  |  |  |  |  |  |  |  |  |  |  |  |  | 47.31 | .005 | |
|  | 18-24 | 4 | 3.2 |  | 6 | 4.7 |  | 4 | 3.1 |  | 2 | 1.7 |  | 3 | 2.3 |  | 11 | 8.0 |  |  |  | |
|  | 25-34 | 7 | 5.6 |  | 9 | 7.1 |  | 7 | 5.3 |  | 11 | 9.2 |  | 10 | 7.7 |  | 5 | 3.6 |  |  |  | |
|  | 35-44 | 10 | 8.1 |  | 13 | 10.2 |  | 20 | 15.3 |  | 23 | 19.2 |  | 13 | 10.0 |  | 18 | 13.0 |  |  |  | |
|  | 45-54 | 13 | 10.5 |  | 27 | 21.3 |  | 13 | 9.9 |  | 15 | 12.5 |  | 22 | 16.9 |  | 14 | 10.1 |  |  |  | |
|  | 55-74 | 65 | 52.4 |  | 54 | 42.5 |  | 73 | 55.7 |  | 42 | 35.0 |  | 56 | 43.1 |  | 71 | 51.4 |  |  |  | |
|  | 75 and over | 25 | 20.2 |  | 18 | 14.2 |  | 14 | 10.7 |  | 27 | 22.5 |  | 26 | 20.0 |  | 19 | 13.8 |  |  |  | |
|  |  |  |  |  |  |  |  |  |  |  |  |  |  |  |  |  |  |  |  |  |  | |
| Race/ethnicity | |  |  |  |  |  |  |  |  |  |  |  |  |  |  |  |  |  |  | 17.40 | .627 | |
|  | White or European | 102 | 82.3 |  | 103 | 81.1 |  | 102 | 77.9 |  | 91 | 75.8 |  | 106 | 81.5 |  | 111 | 80.4 |  |  |  | |
|  | Black or African American | 14 | 11.3 |  | 10 | 7.9 |  | 14 | 10.7 |  | 12 | 10.0 |  | 18 | 13.8 |  | 13 | 9.4 |  |  |  | |
|  | Hispanic or Latina/o/x | 2 | 1.6 |  | 5 | 3.9 |  | 6 | 4.6 |  | 6 | 5.0 |  | 2 | 1.5 |  | 3 | 2.2 |  |  |  | |
|  | Asian or Pacific Islander | 1 | 0.8 |  | 2 | 1.6 |  | 3 | 2.3 |  | 5 | 4.2 |  | 3 | 2.3 |  | 6 | 4.3 |  |  |  | |
|  | Other | 5 | 4 |  | 7 | 5.5 |  | 6 | 4.6 |  | 6 | 5.0 |  | 1 | .8 |  | 5 | 3.6 |  |  |  | |
|  |  |  |  |  |  |  |  |  |  |  |  |  |  |  |  |  |  |  |  |  |  | |
| Education | |  |  |  |  |  |  |  |  |  |  |  |  |  |  |  |  |  |  | 17.50 | .619 | |
|  | High school degree or equivalent (e.g., GED) | 32 | 25.8 |  | 29 | 22.8 |  | 31 | 23.7 |  | 36 | 30.0 |  | 31 | 23.8 |  | 36 | 26.1 |  |  |  | |
|  | Some college but no degree | 32 | 25.8 |  | 40 | 31.5 |  | 38 | 29.0 |  | 28 | 23.3 |  | 25 | 19.2 |  | 38 | 27.5 |  |  |  | |
|  | Associate degree | 18 | 14.5 |  | 13 | 10.5 |  | 20 | 15.3 |  | 17 | 14.2 |  | 21 | 16.2 |  | 23 | 16.7 |  |  |  | |
|  | Bachelor’s degree | 31 | 25 |  | 28 | 22 |  | 25 | 19.1 |  | 20 | 16.7 |  | 34 | 26.2 |  | 30 | 21.7 |  |  |  | |
|  | Graduate degree | 11 | 8.9 |  | 17 | 13.4 |  | 17 | 13.0 |  | 19 | 15.8 |  | 19 | 14.6 |  | 11 | 8.0 |  |  |  | |
|  |  |  |  |  |  |  |  |  |  |  |  |  |  |  |  |  |  |  |  |  |  | |
| Income | |  |  |  |  |  |  |  |  |  |  |  |  |  |  |  |  |  |  | 19.40 | .931 | |
|  | Less than $49,999 | 61 | 49.2 |  | 57 | 44.9 |  | 59 | 45.0 |  | 54 | 45.0 |  | 65 | 50.0 |  | 69 | 50.0 |  |  |  | |
|  | $50,000 to 74,999 | 28 | 22.6 |  | 34 | 26.8 |  | 34 | 26.0 |  | 37 | 30.8 |  | 34 | 26.2 |  | 36 | 26.1 |  |  |  | |
|  | $75,000 to 99,999 | 16 | 12.9 |  | 9 | 7.1 |  | 18 | 13.7 |  | 9 | 7.5 |  | 14 | 10.8 |  | 20 | 14.5 |  |  |  | |
|  | $100,000 to $124,999 | 8 | 6.5 |  | 12 | 9.4 |  | 7 | 5.3 |  | 7 | 5.8 |  | 8 | 6.2 |  | 5 | 3.6 |  |  |  | |
|  | $125,000 to $149,999 | 4 | 3.2 |  | 4 | 3.1 |  | 4 | 3.1 |  | 3 | 2.5 |  | 4 | 3.1 |  | 3 | 2.2 |  |  |  | |
|  | $150,000 to $174,999 | 3 | 2.4 |  | 5 | 3.9 |  | 3 | 2.3 |  | 5 | 4.2 |  | 1 | .8 |  | 3 | 2.2 |  |  |  | |
|  | $175,000 or higher | 4 | 3.2 |  | 6 | 4.7 |  | 6 | 4.6 |  | 5 | 4.2 |  | 4 | 3.1 |  | 2 | 1.4 |  |  |  | |
|  |  |  |  |  |  |  |  |  |  |  |  |  |  |  |  |  |  |  |  |  |  | |
| Region | |  |  |  |  |  |  |  |  |  |  |  |  |  |  |  |  |  |  | 11.99 | .680 | |
|  | West | 32 | 25.8 |  | 28 | 22 |  | 27 | 20.6 |  | 22 | 18.3 |  | 30 | 23.1 |  | 33 | 23.9 |  |  |  | |
|  | South | 50 | 40.3 |  | 38 | 29.9 |  | 45 | 34.4 |  | 39 | 32.5 |  | 42 | 32.3 |  | 44 | 31.9 |  |  |  | |
|  | Northeast | 23 | 18.5 |  | 26 | 20.5 |  | 30 | 22.9 |  | 32 | 36.7 |  | 32 | 24.6 |  | 35 | 25.4 |  |  |  | |
|  | Midwest | 19 | 15.3 |  | 35 | 27.6 |  | 29 | 22.1 |  | 27 | 22.5 |  | 26 | 20.0 |  | 26 | 18.8 |  |  |  | |
|  |  |  |  |  |  |  |  |  |  |  |  |  |  |  |  |  |  |  |  |  |  | |
| Political party | |  |  |  |  |  |  |  |  |  |  |  |  |  |  |  |  |  |  | 14.23 | .163 | |
|  | Democrat | 49 | 40.8 |  | 35 | 28.2 |  | 47 | 38.5 |  | 44 | 37.6 |  | 51 | 42.1 |  | 44 | 33.1 |  |  |  | |
|  | Independent | 28 | 23.3 |  | 45 | 36.3 |  | 35 | 28.7 |  | 27 | 23.1 |  | 28 | 23.1 |  | 47 | 35.3 |  |  |  | |
|  | Republican | 43 | 35.8 |  | 44 | 35.5 |  | 40 | 32.8 |  | 46 | 39.3 |  | 42 | 34.7 |  | 42 | 31.6 |  |  |  | |
|  |  |  |  |  |  |  |  |  |  |  |  |  |  |  |  |  |  |  |  |  |  | |
| Political identity | |  |  |  |  |  |  |  |  |  |  |  |  |  |  |  |  |  |  | 21.21 | .385 | |
|  | Strongly conservative | 20 | 16.5 |  | 23 | 19 |  | 12 | 9.7 |  | 17 | 14.3 |  | 20 | 15.7 |  | 25 | 18.5 |  |  |  | |
|  | Moderately conservative | 38 | 31.4 |  | 33 | 27.3 |  | 31 | 25.0 |  | 33 | 27.7 |  | 29 | 22.8 |  | 30 | 22.2 |  |  |  | |
|  | Neutral | 31 | 25.6 |  | 35 | 28.9 |  | 35 | 28.2 |  | 35 | 29.4 |  | 36 | 28.3 |  | 37 | 27.4 |  |  |  | |
|  | Moderately liberal | 28 | 23.1 |  | 22 | 18.2 |  | 26 | 21.0 |  | 21 | 17.6 |  | 27 | 21.3 |  | 28 | 20.7 |  |  |  | |
|  | Strongly liberal | 4 | 3.3 |  | 8 | 6.6 |  | 20 | 16.1 |  | 13 | 10.9 |  | 15 | 11.8 |  | 15 | 11.1 |  |  |  | |
|  |  |  |  |  |  |  |  |  |  |  |  |  |  |  |  |  |  |  |  |  |  | |
| Disabled | |  |  |  |  |  |  |  |  |  |  |  |  |  |  |  |  |  |  | 8.09 | .151 | |
|  | No | 107 | 86.3 |  | 106 | 83.5 |  | 101 | 77.1 |  | 103 | 85.8 |  | 105 | 80.8 |  | 105 | 76.1 |  |  |  | |
|  | Yes | 17 | 13.7 |  | 21 | 16.5 |  | 30 | 22.9 |  | 17 | 14.2 |  | 25 | 19.2 |  | 33 | 23.9 |  |  |  | |

____________________________________________________________________________________________________________

**Appendix Table A3**

*Scenarios that the participants read*

| **Scenarios and Questions** |
| --- |
| Next, you will be asked to read a description and answer a few questions about an incident at a hypothetical public middle school in the US based on real events. Please consider the following information carefully and answer the questions as a resident of the public school area. |
| **Eight-year-old George/Jamal/Ahanu was kicking his teacher/special education teacher. The teacher/special education teacher physically restrained him to keep him from kicking.**  In school, students are often graded on a scale: A, B, C, D, or F. Using a similar scale, how would you categorize the general job performance of the teacher? |
| **After the incident in class, the principal suspended George/Jamal/Ahanu from school for one day.**  In school, students are often graded on a scale: A, B, C, D, or F. Using a similar scale, how would you categorize the general job performance of the principal? |
| Rate your level of agreement with the following statement:  ***George/Jamal/Ahanu deserved his punishment.*** |
| Rate your level of agreement with the following statement:  ***The punishment fits the crime.*** |
| **After George/Jamal/Ahanu returned from his suspension, he returned to his class and exhibited similar behavior. This time, the principal contacted a sworn police officer.**  In school, students are often graded on a scale: A, B, C, D, or F. Using a similar scale, how would you categorize the general job performance of the principal? |
| **When the sworn police officer arrived to class, George/Jamal/Ahanu tried to punch him. The sworn police officer handcuffed George/Jamal/Ahanu above the elbow for 15 minutes.**  In school, students are often graded on a scale: A, B, C, D, or F. Using a similar scale, how would you categorize the general job performance of the officer? |
| Rate your level of agreement with the following statement:  ***George/Jamal/Ahanu deserved his punishment.*** |
| Rate your level of agreement with the following statement:  ***The punishment fits the crime.*** |
| Rate your level of agreement with the following statement:  ***This incident was prejudiced.*** |

**FIGURES**

***Appendix Figure A1*.** Participant Evaluation of School Personnel Mean Performance Level by Race Experimental Conditions within Scenario.

***Appendix Figure A2*.** Participant Evaluation of School Personnel Mean Performance Level by Disability Experimental Conditions within Scenario.

***Appendix Figure A3*.** Participant Evaluation of Deserve Punishment by Race Experimental Condition within Scenario.

***Appendix Figure A4*.** Participant Evaluation of Deserve Punishment by Disability Experimental Condition within Scenario.

***Appendix Figure A5*.** Participant Evaluation of Punishment Fit the Crime by Scenario within Race Experimental Condition.

***Appendix Figure A6*.** Participant Evaluation of Punishment Fit the Crime by Scenario within Disability Experimental Condition.

***Appendix Figure A7*.** Participant Evaluation of Mean Agreement Level that the Incident was Prejudice by Race Experimental Condition.

***Appendix Figure A8*.** Participant Evaluation of Mean Agreement Level that the Incident was Prejudice by Disability Experimental Condition.

**DisCrit Key Tenets**

(1) DisCrit focuses on ways that the forces of racism and ableism circulate interdependently, often in neutralized and invisible ways, to uphold notions of normalcy.

(2) DisCrit values multidimensional identities and troubles singular notions of identity such as race or dis/ability or class or gender or sexuality, and so on.

(3) DisCrit emphasizes the social constructions of race and ability and yet recognizes the material and psychological impacts of being labeled as raced or dis/abled, which sets one outside of the western cultural norms.

(4) DisCrit privileges voices of marginalized populations, traditionally not acknowledged within research.

(5) DisCrit considers legal and historical aspects of dis/ability and race and how both have been used separately and together to deny the rights of some citizens.

(6) DisCrit recognizes whiteness and Ability as Property and that gains for people labeled with dis/abilities have largely been made as the result of interest convergence of white, middle-class citizens.

(7) DisCrit requires activism and supports all forms of resistance.

**Recommendations for Change Based on Prior Research**

Here, multiple recommendations for change based on prior research have been listed. First, there is a keen need to advance legislation that offers protections and safeguards against restraint and seclusion. For example, mandating at the state and federal level that staff must periodically check on secluded or restrained children. Even when policies fail to advance, there are related policy changes that happen at the state or district level with, for example, states strengthening their safeguards for restraint and seclusion (Butler, 2019). Secondly, there is a critical need for regulatory requirements to document, report, and review incidents of restraint and seclusion in schools (Bartlett & Ellis, 2021)—especially for children with disabilities who may not be able to communicate what happened on their own (Butler, 2019).

Moreover, there is also a critical need for implicit bias training to disrupt anti-disabled bias among school personnel. School personnel are 20 times more likely to restrain and seclude disabled students than non-disabled students (CRDC, 2012)—which indicates some level of discrimination based on disability. Mandating implicit bias trainings as part of licensure requirements is a critical first step, and it could help address other types of bias like racial bias. Studies show that persons rely on implicit biases unconsciously when under duress (Greenwald & Krieger, 2006; Kang et al., 2011) which could increase the chances of school personnel employing dangerous practices like restraint and seclusion at a disproportionally high rates for disabled students.

**References**

Bartlett, N. A., and Ellis, T. F. (2021). Policies matter: closing the reporting and transparency

gaps in the use of restraint, seclusion, and time-out rooms in schools. Can. J. Educ. Adm.

Policy 196, 2–15. doi: 10.7202/1078514ar

Butler, J. (2019). How safe is the schoolhouse? An analysis of state seclusion and restraint laws

and policies. Available online at: https://mail.autcom.org/public/assets/

HowSafeSchoolhouse.pdf

CRDC 2012. Revealing new truths about our nation’s schools. Available online at:

http://ocrdata.ed.gov/Downloads/CMOCRTheTransformedCRDCFINAL3-15-12Acce

ssible-1.pdf

Greenwald, A. G., and Krieger, L. H. (2006). Implicit bias: scientific foundations. Calif. Law

Rev. 94, 945–967. doi: 10.2307/20439056

Kang, J., Bennett, M., Carbado, D., Casey, P., and Levinson, J. (2011). Implicit bias in the

courtroom. UCLA Law Rev. 59:1124.
